# Prenatal alcohol exposure affects brain function during place learning in a virtual environment differently in boys and girls

**DOI:** 10.1002/brb3.1103

**Published:** 2018-10-23

**Authors:** Keri J. Woods, Kevin G. F. Thomas, Christopher D. Molteno, Joseph L. Jacobson, Sandra W. Jacobson, Ernesta M. Meintjes

**Affiliations:** ^1^ Division of Biomedical Engineering, Department of Human Biology Faculty of Health Sciences, University of Cape Town South Africa; ^2^ UCT Neuroscience Institute, Faculty of Health Sciences, University of Cape Town South Africa; ^3^ Department of Psychology University of Cape Town South Africa; ^4^ Department of Psychiatry and Mental Health Faculty of Health Sciences, University of Cape Town South Africa; ^5^ Department of Psychiatry and Behavioral Neurosciences Wayne State University School of Medicine Detroit Michigan; ^6^ Cape Universities Body Imaging Centre University of Cape Town South Africa

**Keywords:** fetal alcohol spectrum disorders, functional magnetic resonance imaging, parahippocampal gyrus, prenatal alcohol exposure, sex differences, spatial navigation

## Abstract

**Introduction:**

Although performance deficits in place learning have been reported in fetal alcohol spectrum disorders (FASD), neural correlates of these deficits have not been investigated. This functional magnetic resonance imaging (fMRI) study of 57 children (41 alcohol‐exposed; 16 controls; mean age = 9.4 years; 29 boys) examined effects of prenatal alcohol exposure (PAE) on place learning in a virtual environment, the computer‐generated (CG) arena.

**Methods:**

Functional magnetic resonance imaging data were acquired while children passively viewed a recording of an experimenter completing the task. Visible‐target blocks involved navigation to a visible platform. During invisible‐target blocks, the platform appeared only when the experimenter moved over it. After the scan, all children performed a post‐test during which they had to navigate to the location of the invisible platform.

**Results:**

Although there were no group differences in post‐test performance for sex or FASD diagnosis, PAE in boys was associated with poorer performance and reduced activation in the parahippocampal gyrus (PHG), precuneus, posterior cingulate, frontal and temporal lobes, caudate, insula, claustrum, lentiform nucleus, and thalamus. By contrast, PAE was not associated with performance or activation in any regions in girls.

**Discussion and conclusion:**

Girls and boys are known to use different navigation strategies. Boys rely more on an allocentric navigational strategy and girls more on landmarks. Poorer recruitment of the PHG, a region known to mediate allocentric navigation, in more heavily exposed boys may explain the observed dose‐dependent place learning deficit. The absence of PAE effects in girls suggests that landmark‐based navigational strategies may be less affected by alcohol exposure.

## 1 INTRODUCTION

Prenatal alcohol exposure (PAE) is associated with impairment in brain structure and function that may lead to cognitive, behavioral, and neurological deficits of variable severity (Archibald, et al., 2001; Astley, Aylward, et al., 2009; Meintjes, et al., 2014; Riley & McGee, 2005; Sowell, et al., 2001). Fetal alcohol syndrome (FAS), the most severe of the fetal alcohol spectrum disorders (FASD), is characterized by small head circumference, pre‐ and/or postnatal growth retardation, and characteristic facial features, including short palpebral fissures, thin vermillion, and flat philtrum (Hoyme, et al., 2005). Two of the three facial features are also seen in partial FAS (PFAS), together with either small head circumference, retarded growth, or neurobehavioral deficits. Heavily exposed (HE) individuals who lack the distinctive pattern of FAS dysmorphology are diagnosed with alcohol‐related neurodevelopmental disorder (ARND) if they exhibit cognitive and/or behavioral impairment (Hoyme, et al., 2005; Stratton, Howe, & Battaglia, 1996).

Cognitive deficits associated with PAE include lower IQ (Jacobson, Jacobson, Sokol, Chiodo, & Corobana, 2004; Streissguth, Barr, & Sampson, 1990), poor attention and executive function (Burden, Jacobson, Sokol, & Jacobson, 2005; Coles, et al., 1997; Kodituwakku, Handmaker, Cutler, Weathersby, & Handmaker, 1995; Mattson, Goodman, Caine, Delis, & Riley, 1999; Rasmussen, 2005), impaired learning and memory (Crocker, Vaurio, Riley, & Mattson, 2011; Lewis, et al., 2015; Mattson & Roebuck, 2002; Roebuck‐Spencer & Mattson, 2004; Vaurio, Riley, & Mattson, 2011), arithmetic difficulties (Burden, Jacobson, & Jacobson, 2005; Chiodo, Jacobson, & Jacobson, 2004; Coles, et al., 1991; Jacobson, et al., 2004; Streissguth, et al., 1994, 1990; Woods, Meintjes, Molteno, Jacobson, & Jacobson, 2015), slower cognitive processing speed (Coles, Platzman, Lynch, & Freides, 2002; Jacobson, Jacobson, & Sokol, 1994; Jacobson, Jacobson, Sokol, Martier, & Ager, 1993; Streissguth, et al., 1990), and compromised visual–spatial ability (Astley, Olson, et al., 2009; Mattson & Riley, 1998; Rasmussen, Horne, & Witol, 2006).

The two distinct components of spatial navigation, spatial location memory and place learning, are both affected by PAE. Impairment in spatial location memory has been demonstrated using tabletop tests (Kaemingk & Tanner Halverson, 2000; Uecker & Nadel, 1996, 1998), the visual learning task from the wide range assessment of memory and learning (WRAML) (Kaemingk, Mulvaney, & Halverson, 2003; Mattson & Roebuck, 2002; Sheslow & Adams, 1990), and spatial *n*‐back tasks (Malisza, et al., 2005, 2012; Norman, et al., 2013). PAE‐related place learning deficits have been shown in rodents and in humans. In the Morris water maze (MWM; Morris, 1981), ethanol‐exposed rodents took longer than unexposed controls to learn escape routes (Blanchard, Riley, & Hannigan, 1987; Gianoulakis, 1990; Richardson, Byrnes, Brien, Reynolds, & Dringenberg, 2002). In computer‐simulated versions of the MWM (Astur, Ortiz, & Sutherland, 1998), children prenatally exposed to alcohol performed more poorly during a probe trial (Mattson, et al., 2010) and travelled greater distances to reach a hidden platform (Hamilton, Kodituwakku, Sutherland, & Savage, 2003; Meintjes, et al., 2012).

Functional MRI (fMRI) and positron emission tomography (PET) studies have identified the hippocampus and parahippocampal gyrus, precuneus, retrosplenial cortex, posterior and inferior parietal cortices, intraparietal sulcus, fusiform gyrus, lingual gyrus, caudate nucleus, thalamus, prefrontal areas, and cerebellum as key regions for navigation (Burgess, Maguire, & O'Keefe, 2002; Constantinidis & Wang, 2004; Curtis, 2006; Grön, Wunderlich, Spitzer, Tomczak, & Riepe, 2000; Halligan, Fink, Marshall, & Vallar, 2003; Jordan, Heinze, Lutz, Kanowski, & Jäncke, 2001; Jordan, Wüstenberg, Heinze, Peters, & Jäncke, 2002; Lamm, Windischberger, Leodolter, Moser, & Bauer, 2001; Maguire, Burgess, & O'Keefe, 1999; McNaughton, Chen, & Markus, 1991; Pine, et al., 2002; Ricciardi, et al., 2006; Shelton & Gabrieli, 2002; Spiers & Barry, 2015; Thomsen, et al., 2000). One hypothesis that links these disparate regions proposes that the posterior parietal and retrosplenial cortices are involved in the translation between allocentric representations in the parahippocampal gyrus and egocentric representations in the medial parietal region (Bird & Burgess, 2008; Burgess & el al., 2001).

Although the hippocampus is recognized as being essential for successful place learning by rodents in the MWM (Brandeis, Brandys, & Yehuda, 1989; Morris, Garrud, Rawlins, & O'Keefe, 1982; Morris, Hagan, & Rawlins, 1986), as well as for successful place learning by humans in virtual versions of the MWM and other virtual environments (Astur, Taylor, Mamelak, Philpott, & Sutherland, 2002; Bohbot, Iaria, & Petrides, 2004; Spiers, et al., 2001), the parahippocampal gyrus, posterior parietal regions (especially the precuneus), fusiform gyrus, and thalamus (Parslow, et al., 2004; Shipman & Astur, 2008) also play a role.

Sex differences in spatial navigation have been widely reported, with males performing better across a range of ages, including young adulthood (Astur, et al., 1998; Astur, Tropp, Sava, Constable, & Markus, 2004; Burkitt, Widman, & Saucier, 2007; Driscoll, Hamilton, Yeo, Brooks, & Sutherland, 2005; Mueller, Jackson, & Skelton, 2008; Nowak, Diamond, Land, & Moffat, 2014; Sneider, et al., 2015; van Gerven, Schneider, Wuitchik, & Skelton, 2012; Woolley, et al., 2010), older adulthood (Driscoll, et al., 2005), prepubertal childhood (Newhouse, Newhouse, & Astur, 2007), and adolescence (Sneider, et al., 2015). Notably, some fMRI studies have shown differences in brain activation between males and females during spatial navigation (Grön, et al., 2000; Wunderlich, 2001), even in the absence of performance differences (Sneider, Sava, Rogowska, & Yurgelun‐Todd, 2011). In those that found sex differences, females activated the right inferior parietal lobule, right superior parietal lobule, left superior and right medial frontal gyri, and the right prefrontal cortex more than males (Grön, et al., 2000; Wunderlich, 2001), while males showed greater activation of the right and left parahippocampal gyri, left hippocampus, and left posterior cingulate (Grön, et al., 2000; Wunderlich, 2001). These sex differences in performance and patterns of brain activation suggest that males and females may use different navigation strategies.

Of particular interest for this paper is that in a T‐maze spatial task, both male and female rats from the PAE group showed reference memory deficits, while working memory was impaired only in the PAE male rats (Zimmerberg, Sukel, & Stekler, 1991). Consistent with this finding, Hamilton et al. (2003) reported alcohol‐related deficits in place learning in boys, although that study did not include any girls. These findings, combined with the known sex differences in strategy and brain activation during navigation, suggest that navigation in males and females may be differentially affected by PAE.

To our knowledge, this is the first study to examine effects of PAE on neural correlates of place learning. This study includes children with FAS and PFAS as well as nonsyndromal HE children. We also examine the association between navigation performance and brain activation during navigation with continuous measures of PAE and investigate whether there are sex differences in the effects of PAE on performance and brain activation during navigation. We hypothesized that alcohol‐exposed children would perform more poorly than controls and would also show altered patterns of brain activation. Further, we predicted that boys would perform better than girls on this task and that there would be sex differences in alcohol‐related alterations in brain activation during navigation.

## 2 METHODS

### 2.1 Participants

Participants were 57 right‐handed 8‐ to 10‐year‐old children from the Cape Coloured (mixed ancestry) community in Cape Town, South Africa. Forty‐one of these children had been heavily exposed to alcohol prenatally (Jacobson, et al., 2008). The Cape Coloured community is composed primarily of descendants of white European settlers, Malaysian slaves, Khoi‐San aboriginals, and black African ancestors. The incidence of FASD in this population is exceptionally high due to poor socioeconomic circumstances and historical practices of compensating farm laborers with wine, both of which have contributed to a tradition of heavy recreational weekend binge drinking (May, et al., 2013, 2007 ).

### 2.2 Procedure

The children's mothers were recruited between 1999 and 2002 at their first visit to the antenatal clinic. Each woman was interviewed, using a timeline follow‐back approach adapted to reflect how pregnant women in this community drink (Jacobson, Chiodo, Sokol, & Jacobson, 2002; Jacobson, et al., 2008; Sokol, et al., 1985), regarding her alcohol consumption during pregnancy. At recruitment, the mother was interviewed regarding the incidence and amount of her drinking on a day‐by‐day basis during a typical 2‐week period at the time of conception. She was also asked whether her drinking had changed since conception; if so, when the change had occurred, and how much she had drunk on a day‐by‐day basis during the preceding 2‐week period. This procedure was repeated in midpregnancy and again at 1 month postpartum to provide information about drinking during the latter part of pregnancy. Volume was recorded for each type of beverage consumed each day, converted to ounces of absolute alcohol (AA) using multipliers proposed by Bowman, Stein, and Newton (1975), and averaged to provide three continuous measures of alcohol consumption around time of conception and during pregnancy: average ounces of AA consumed/day, AA/occasion, and frequency of drinking (days/week).

Two groups of women were recruited: (a) heavy drinkers, who consumed at least 14 standard drinks per week (1.0 oz AA⁄day) on average or who engaged in binge drinking (5 or more drinks/occasion) and (b) controls whose mothers abstained or drank no more than minimally during pregnancy. The number of cigarettes smoked/day was also recorded, as was the use of illicit drugs (days/week). Mothers were also interviewed regarding their age at delivery, education (years completed), and marital status.

In September 2005, we organized a clinic in which each child was independently examined for growth and FAS dysmorphology by two expert FAS dysmorphologists (H.E. Hoyme, MD, and L.K. Robinson, MD) using the Hoyme et al. (2005) protocol (see Jacobson, et al., 2008). A subset of children who could not attend the clinic was examined by another FAS dysmorphologist (N. Khaole, MD). There was substantial agreement among the examiners on the assessment of all dysmorphic features, including the three principal fetal alcohol‐related features—philtrum and vermilion (which were measured on the Astley and Clarren (2001) rating scales) and palpebral fissure length (*r*s = 0.80, 0.84, and 0.77, respectively; kappas = 0.87, 0.80, and 0.78, respectively). FAS and PFAS diagnoses were agreed upon at a case conference by the dysmorphologists (HEH and LKR), SWJ, JLJ, and CDM. Eight children met the Hoyme et al. (2005) criteria for full FAS; 19 did for PFAS. The 14 alcohol‐exposed children who did not meet criteria for either FAS or PFAS were designated nonsyndromal HE.

Written informed consent was obtained from each mother and written assent from each child. Approval for the research procedures was obtained from the Wayne State University and UCT Faculty of Health Sciences Human Research Ethics Committees.

### 2.3 Neuropsychological assessment

IQ data were collected from the children on the Wechsler Intelligence Scale for Children‐IV (WISC‐IV) at 10 years (Diwadkar, et al., 2013; Jacobson, et al., 2011). At the 5‐year follow‐up of these children, we administered the Junior South African Individual Scales (JSAIS; Madge, Berg, & Robinson, 1981), which are available in Afrikaans and in English and have been normed for South African children. IQ scores from the JSAIS were strongly correlated with the WISC‐IV scores for the children in this sample, *r = *0.74, *p* < 0.001, supporting the validity of the WISC for use with this population.

### 2.4 Neuroimaging assessment

#### 2.4.1 Magnetic resonance imaging protocol

All scans were acquired using a 3 T Allegra MRI scanner (Siemens Medical Systems, Erlangen, Germany). High‐resolution anatomic images were acquired in the sagittal plane using a three‐dimensional magnetization‐prepared rapid gradient echo sequence (160 slices, TR = 2,300 ms, TE = 3.93 ms, TI = 1,100 ms, slice thickness 1 mm, resolution 1.3 × 1.0 × 1.0 mm^3^). During the fMRI protocol, 213 functional volumes sensitive to blood oxygen level‐dependent contrast were acquired with a T2*‐weighted gradient echo, echo planar imaging sequence (TR = 2,000 ms, TE = 30 ms, 34 interleaved slices, 3 mm thick, 200 × 200 mm^2^ field of view, resolution 3.125 × 3.125 × 3 mm^3^).

#### 2.4.2 Functional MRI experimental tasks

We used a virtual navigation environment known as the computer‐generated (CG) arena (Jacobs, Laurance, & Thomas, 1997; Jacobs, Thomas, Laurance, & Nadel, 1998; Thomas, Hsu, Laurance, Nadel, & Jacobs, 2001), which presents tasks similar in form and appearance to those presented by the virtual water maze task used in the Hamilton et al. (2003) study. All participants practiced the task using a desktop‐based version of the CG arena before scanning and also listened to a recording of the scanner noises while lying in a mock scanner. During the scan, the CG arena task was presented using a data projector positioned in a room behind the scanner. Images were projected through a waveguide in line with the bore of the magnet onto a rear projection screen mounted behind the scanner, which the children viewed using the standard mirror system that mounts to the head coil. Auditory tones were presented using the standard Siemens headphones. The children were able to talk to the examiner using an intercom that is built into the scanner. Children could stop the scan at any time by squeezing a ball held in the left hand.

**Figure 1:**
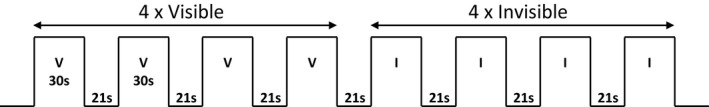
Timing diagram of the CG arena task

**Figure 2:**
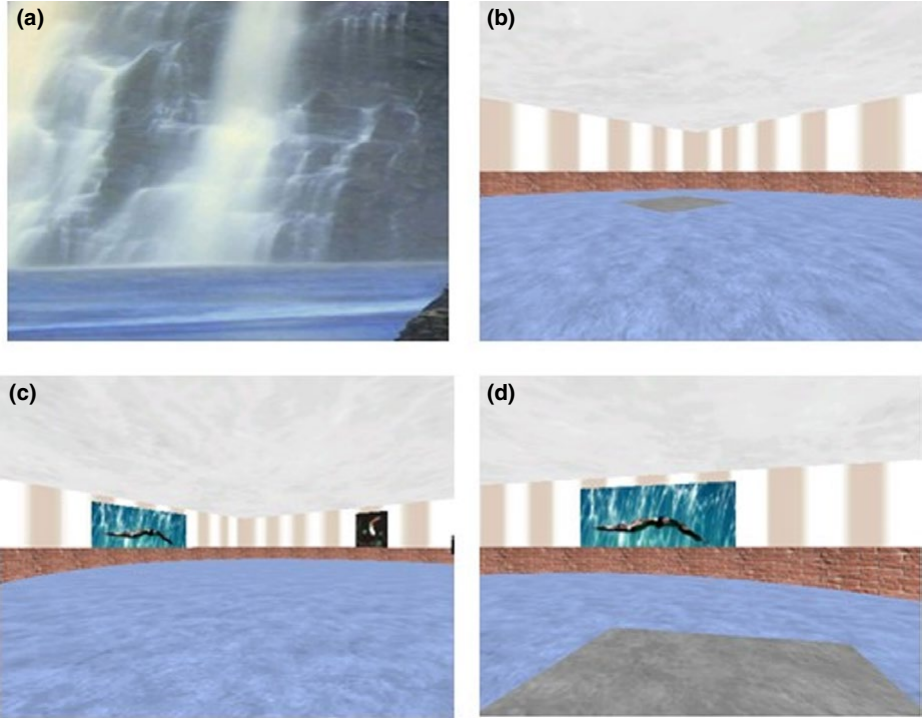
(a) Picture displayed during rest blocks; (b) example of a visible platform in a room with no pictures; (c) example of a room with an invisible platform; and (d) the platform appearing when the subject moves over it

Children were scanned while passively viewing a recording of another person navigating around the CG arena room to the platform, which was sometimes visible (visible‐target condition) and sometimes hidden (invisible‐target condition). The task comprised four repetitions of the visible condition (duration 30 s each), followed by four repetitions of the invisible condition (duration 30 s each), with 21‐s rest blocks between each active block (Figure 1). An initial 19‐s rest block preceded the first visible block, and a final 20‐s rest block followed the last invisible block (total task duration 426 s). During rest blocks, a static picture of a waterfall was displayed (Figure 2a). During visible‐target blocks, a gray platform was visible on the floor of a circular brick enclosure in a square room (Figure 2b). To prevent subjects from learning the room during visible conditions, there were no pictures on the walls and the location of the platform changed for each repetition of the visible block. During invisible‐target blocks, the platform remained hidden (Figure 2c) until the person navigating moved over it (Figure 2d). The location of the platform remained the same in each repetition of the invisible condition. Hence, the aim was for the subject to learn the location of the invisible platform using distal cues within the room, such as pictures on the walls.

#### 2.4.3 Behavioral performance

After completing the scan, the children performed a post‐scan test in a small room adjacent to the scanner. The purpose of this test was to confirm that they had learnt the location of the platform during the invisible‐target condition. The test, which comprised two 120‐s trials, involved using a joystick to navigate within the CG arena to the location of the invisible platform. Time to navigate to the location of the platform (“latency”) and length of path to that location were used to assess task performance. Because the post‐scan assessment indicated that all participants had been attending to the task, none of the children were excluded based on performance.

#### 2.4.4 fMRI analysis

All fMRI analyses were performed using Brain Voyager QX (Brain Innovation, Maastricht, The Netherlands). Four dummy images were acquired that were excluded from all analyses. Images were motion‐corrected relative to the first volume with trilinear/sinc interpolation. Images were corrected for different slice acquisition times and linear trends and temporally smoothed with a high‐pass filter of two cycles/point.

Because the present data were acquired near the end of the imaging session, the data contained substantial motion artifacts. For each subject, data from the largest continuous section with no movement greater than 3 mm displacement or 3.0° rotation were analyzed. Children were only included in the analysis if they had usable data from at least one block for each condition.

Each child's functional data were coregistered to his/her high‐resolution anatomic MRI, rotated into the AC‐PC plane, and normalized to Talairach space using a linear transform calculated on the anatomic images. The 3.125 × 3.125 × 3 mm^3^ fMRI voxels were interpolated during Talairach normalization to 3 × 3 × 3 mm^3^.

Whole brain group analyses were performed with a random effect analysis of variance (ANOVA) using a generalized linear model (GLM) with predictors based on the visible‐target and invisible‐target experimental blocks convolved by the standard hemodynamic function. The six motion correction parameters were *z*‐transformed and added as predictors of no interest. Beta maps were created for each subject for each condition of interest (i.e., visible target and invisible target). To examine whether the difference in activation between the invisible‐target and visible‐target conditions differed by sex, the beta maps were analyzed at the second level using a repeated‐measures ANOVA, with one within‐subjects factor (i.e., invisible target vs. visible target) and one between‐subjects factor (i.e., sex). The voxelwise threshold was set to *p < *0.01, with cluster‐size thresholding to control for multiple comparisons using the Monte Carlo simulation tool implemented in Brain Voyager (Forman, et al., 1995); minimum cluster size = 324 mm^3^. The analysis was repeated controlling for PAE.

Because previous studies have found that males and females activate different regions during navigation (Grön, et al., 2000; Sneider, et al., 2011; Wunderlich, 2001), we identified regions separately in boys and girls where differences in activation between invisible‐ and visible‐target conditions were associated with extent of PAE. Beta maps were created for each subject for the invisible‐ versus visible‐target contrast and analyzed separately for boys and girls at the second level in a GLM in relation to AA/day. The voxelwise threshold was set to *p < *0.05, with cluster‐size thresholding (minimum cluster size =1,026 mm^3^). In each identified cluster, mean % signal change was extracted for each subject for each condition to examine in each region whether the association of degree of PAE with the difference in activation between invisible‐target and visible‐target conditions survives after controlling for potential confounders.

To identify regions crucial for successful place learning, we repeated the second‐level analysis, again separately for boys and girls, with path length and time to target, respectively, as continuous outcome measures. The voxelwise threshold was set to *p* < 0.05, with cluster‐size thresholding (minimum cluster size = 1,107 and 1,134 mm^3^ for path length and latency, respectively). Mean % signal change values were extracted in the clusters for each subject to examine whether associations survive after controlling for alcohol exposure.

### 2.5 Statistical analyses

All variables were examined for normality of distribution. The following variables with outliers greater than 3 standard deviations (*SD*) beyond the mean were transformed by recoding all outlying values to 1 point beyond the highest/lowest observed value: AA/day across pregnancy (*n = *1), smoking during pregnancy (*n = *1), mother's education (*n = *1), postnatal lead exposure (*n = *1), mean % signal change differences between invisible and visible conditions in the clusters centered on the right middle frontal gyrus and the bilateral precuneus where boys showed associations with AA/day (*n = *1 each), mean % signal change differences between invisible and visible conditions in the clusters centered on the bilateral precuneus and the left middle occipital gyrus where boys showed greater activation than girls (*n = *1 each), path length to target (*n = *2), and latency (*n = *1).

Six control variables were assessed for consideration as potential confounders of the relation of PAE to spatial navigation: two maternal demographic characteristics (mother's age at delivery and years of education), two child characteristics (sex and age at assessment), and two other exposures (maternal smoking during pregnancy and postnatal lead exposure) known to impact on the child's academic performance. Lead exposure, based on a venous blood sample obtained from the child at 5 years, was included because lead levels in this population are within the range in which subtle but meaningful effects on cognitive function have consistently been reported (e.g., Chiodo, et al., 2004; Lanphear, Dietrich, Auinger, & Cox, 2000). Each control variable that was even weakly related (at *p* < 0.20) to a given outcome measure was considered a potential confounder of the effect of alcohol exposure on the outcome in question.

The outcome measures were latency (in seconds), path length to reach the target (in arena units), and difference in % signal change between the invisible and the visible conditions in each of the regions where associations with extent of PAE were found for boys and girls separately. Differences between diagnostic groups (FAS/PFAS; HE; control) were examined for each of the behavioral measures using analysis of variance (ANOVA). *Post hoc* comparisons were computed using the least‐squares difference (LSD) approach. Sex differences in each of the behavioral measures were examined using *t*‐tests. Sex differences in performance were also examined within each diagnostic group. ANCOVA was used to examine whether effects remained significant after controlling for potential confounders related to the outcome in question at *p* < 0.20. The relation of the continuous measure of PAE across pregnancy, AA/day, to each of the behavioral measures was examined using Pearson correlation analysis, for all the children combined and for boys and girls separately. Multiple regression analysis relating the continuous exposure measure to each of the outcomes was used to adjust for potential confounding by control variables related to the outcome in question at *p < *0.20.

All analyses were repeated omitting those children whose mothers used illicit drugs during pregnancy.

## 3 RESULTS

### 3.1 Sample characteristics

Fifty‐seven children were scanned: 27 with FAS or PFAS, 14 HE, and 16 controls. Sample characteristics for these children are summarized in Table 1. Mothers of children with FAS/PFAS reported having consumed an average of 7.8 standard drinks of alcohol per drinking occasion during pregnancy, in contrast to the average of 5.2 for mothers of the HE group. Furthermore, the mothers of the FAS/PFAS group consumed alcohol about twice as often as the HE mothers. All but one of the control mothers abstained from drinking during pregnancy; the one light‐drinking control mother consumed 2 drinks on 3 occasions.

**Table 1:** Sample characteristics (*N = *57)

	FAS/PFAS (*n = *27)	Heavily exposed Nonsyndromal (*n = *14)	Control (*n = *16)	*F* or χ^2^ (*p*)
Maternal				
Age at delivery	29.0 (7.4)	25.5 (5.0)	25.7 (3.3)	2.37 (0.103)
Education (years)^a^	7.1 (2.2)	8.6 (3.1)	10.6 (1.6)	**11.49 (0.001)**
Marital status (% married)	44.4	35.7	68.8	3.71 (0.156)
Smoking during pregnancy (cigarettes/day)	8.0 (5.7)	8.4 (7.2)	3.4 (9.9)	2.36 (0.104)
Prenatal alcohol exposure				
AA/day at conception (oz)^b^	1.8 (2.1)	0.6 (0.6)	0.0 (0.0)	**7.18 (0.002)**
AA/occasion at conception (oz)^c^	4.3 (2.5)	2.6 (2.5)	0.1 (0.3)	**19.57 (*<*0.001)**
Frequency at conception (days/week)^d^	2.6 (1.7)	1.3 (1.1)	0.0 (0.1)	**21.00 (<0.001)**
AA/day across pregnancy (oz)^e^	1.2 (1.4)	0.5 (0.5)	0.0 (0.0)	**7.66 (0.001)**
AA/occasion across pregnancy (oz)^f^	3.9 (1.9)	2.6 (1.6)	0.1 (0.3)	**30.40 (<0.001)**
Frequency across pregnancy (days/week)^g^	2.0 (1.4)	1.1 (0.9)	0.0 (0.0)	**18.13 (<0.001)**
Child				
Sex (% male)	51.9	50.0	50.0	0.02 (0.990)
Age at scan	9.4 (0.3)	9.6 (0.6)	9.4 (0.4)	2.31 (0.109)
Blood lead concentration (μg/dl)^h^	11.9 (9.5)	9.5 (3.9)	7.9 (3.0)	**3.39 (0.041)**
WISC‐IV‐Full Scale IQ^i^	64.5 (9.5)	72.8 (8.2)	76.4 (9.1)	**9.43 (<0.001)**
Behavioral performance				
Path length to target (arena units within a 500 X 500 grid)	140.1 (130.1)	148.6 (99.0)	118.3 (132.5)	0.25 (0.780)
Latency (s)	31.1 (31.7)	27.1 (19.9)	20.8 (20.6)	0.76 (0.473)

Notes

AA: absolute alcohol; 1 oz AA ≈ two standard drinks; FAS: fetal alcohol syndrome; PFAS: partial FAS; WISC‐IV: Wechsler Intelligence Scale for Children‐Fourth Edition.

Values are mean (*SD*); BOLD font denotes significance at *p* < 0.05.

a FAS/PFAS < cont, *p < *0.0001; HE < cont, *p* = 0.027; FAS/PFAS < HE, *p* = 0.044.

b Cont < FAS/PFAS, *p = *0.001; cont < HE, *p* = 0.266; HE < FAS/PFAS, *p = *0.29.

c Cont < FAS/PFAS, *p < *0.0001; cont < HE, *p = *0.002; HE < FAS/PFAS, *p = *0.017.

d Cont < FAS/PFAS, *p < *0.0001; cont < HE, *p = *0.011; HE < FAS/PFAS, *p = *0.002.

e Cont < FAS/PFAS, *p < *0.0001; cont < HE, *p* = 0.178; HE < FAS/PFAS, *p = *0.036.

f Cont < FAS/PFAS, *p* < 0.0001; cont < HE, *p* < 0.0001; HE < FAS/PFAS, *p = *0.016.

g Cont < FAS/PFAS, *p < *0.0001; cont < HE, *p = *0.005; HE < FAS/PFAS, *p = *0.015.

h Cont < FAS/PFAS, *p = *0.014.

i FAS/PFAS < cont, *p < *0.0001; FAS/PFAS < HE, *p = *0.008.

As expected, the groups differed in alcohol exposure but were generally similar otherwise, with a few exceptions. These exceptions included the following: (a) mothers of children with FAS/PFAS had less formal education than the other two groups, and mothers of the HE group had fewer years of education than mothers of controls; (b) on average, children with FAS/PFAS had greater lead concentrations than controls; and (c) as expected, children with FAS/PFAS scored more poorly on the WISC‐IV than HE and control children. Regarding illicit drug use within the sample, three mothers of children with FAS/PFAS reported using marijuana during pregnancy, and one mother of a child with FAS/PFAS reported using cocaine.

After exclusions due to excessive motion, 41 children provided usable functional data: 19 with FAS/PFAS (nine boys and ten girls), 10 HE (four boys and six girls), and 12 controls (six boys and six girls). Mean and maximum displacements did not differ between diagnostic groups (*F*(2, 38) = 0.25, *p* = 0.78 and *F*(2, 38) = 0.91, *p* = 0.41, respectively). The children with usable scanner data did not differ from those excluded in terms of alcohol exposure (*t*(55) = 1.29, *p = *0.20 for AA/day), FASD diagnostic group (χ^2^(2, *N = *57) = 0.11, *p = *0.95), CG arena task performance (all *p*s > 0.90), or any other demographic variable (all *p*s > 0.20).

### 3.2 Behavioral performance

All 57 children in the sample were included in the behavioral analyses. We only report data from the first trial of the post‐scan navigation test because performance on that trial best reflects whether the child learnt the location of the platform during the scan. Performance on the second post‐scan trial, in contrast, is contaminated by any learning that might take place during the first trial. All children had practiced the task extensively before scanning and hence were comfortable with the procedure.

Among all children, as well as within boys and girls separately, neither path length nor latency differed between diagnostic groups (Table 2). Boys and girls performed similarly, both for the sample as a whole and within each diagnostic group (all *p*s > 0.20, Table 2). Path length to target and latency were also not related (at *p* < 0.20) to any of the other control variables (Table 3), except for lead exposure.

**Table 2:** Comparison of post‐test performance by sex and diagnostic group

	All (*N* = 57)	Boys (*N* = 29)	Girls (*N* = 28)	*t* (*p)*
Path length	130.1 (104.1)	137.0 (108.3)	122.9 (101.1)	0.51 (0.614)
Latency	26.8 (24.8)	26.0 (26.5)	27.6 (23.4)	0.24 (0.809)
Within‐ and between‐group differences				
Path length				
FAS/PFAS (*N* = 27; 14 boys)	133.4 (110.5)	156.6 (127.0)	108.5 (87.7)	1.13 (0.267)
HE (*N* = 14; 7 boys)	148.6 (99.1)	138.5 (83.9)	158.7 (118.2)	0.37 (0.719)
Control (*N* = 16; 8 boys)	108.2 (99.8)	101.5 (93.2)	115.0 (112.0)	0.26 (0.797)
Between‐group ANOVA *F* (*p*)	0.58 (0.564)	0.64 (0.534)	0.58 (0.570)	
Latency				
FAS/PFAS (*N* = 27; 14 boys)	30.1 (29.1)	34.3 (34.4)	25.6 (22.7)	0.76 (0.453)
HE (*N* = 14; 7 boys)	27.1 (19.9)	20.1 (17.3)	34.0 (21.1)	1.35 (0.202)
Control (*N* = 16; 8 boys)	20.8 (20.6)	16.6 (8.8)	25.1 (28.2)	0.82 (0.428)
Between‐group ANOVA *F* (*p*)	0.70 (0.503)	1.40 (0.265)	0.34 (0.717)	

Note

FAS: fetal alcohol syndrome; PFAS: partial FAS; HE: heavily exposed nonsyndromal.

Values are mean (*SD*).

**Table 3:** Correlation between control variables and behavioral measures for all children (*N = *57)

	Child's age at scan	Lead exposure	Maternal age	Maternal education	Smoking during pregnancy
Path length to target	−0.05 (0.717)	0.30 (0.025)	0.08 (0.574)	−0.16 (0.239)	0.02 (0.862)
Latency	−0.15 (0.258)	0.23 (0.085)	0.06 (0.647)	−0.16 (0.234)	−0.02 (0.884)

Values are Pearson r(p).

The relation of PAE to behavioral performance is shown in Table 4, both before and after controlling for lead exposure. There was an overall effect of PAE on latency, largely due to the effect within the boys. In boys, increased alcohol exposure was associated with both longer path length to target and longer latency to target. The relation between alcohol exposure and latency to target remained significant after controlling for lead exposure. In girls, there were no significant relations between performance and alcohol exposure levels.

**Table 4:** Relation of prenatal alcohol exposure to behavioral performance

	AA per day (oz)	
	*r* (*p*)	ß^a^ (*p*)
All (*N = *57)		
Path length to target	0.21 (0.113)	0.14 (0.288)
Latency	0.26 (0.051)	0.21 (0.119)
Boys (*N* = 29)		
Path length to target	**0.38 (0.044)**	0.30 (0.142)
Latency	**0.50 (0.006)**	**0.47 (0.018)**
Girls (*N* = 28)		
Path length to target	0.11 (0.584)	0.06 (0.755)
Latency	0.08 (0.699)	0.04 (0.832)

Note

AA: absolute alcohol; bold font denotes significance at *p* < 0.05.

a Adjusted for lead exposure.

### 3.3 Neuroimaging assessments

Boys showed greater increases than girls in activation during the invisible condition compared to the visible condition in the right middle frontal gyrus and inferior parietal lobule, and left precuneus, superior parietal lobule, lingual gyrus, middle occipital gyrus, and middle temporal gyrus (Table 5). Sex differences in activation remained significant after controlling for PAE. There were no regions where girls showed greater differences than boys in activation between the two conditions. Whereas boys showed increased activation in the invisible‐target compared to visible‐target condition, girls showed reduced activation during the invisible‐target condition in diffuse regions across all lobes.

**Table 5:** Regions where the difference in activation between the invisible and visible conditions is greater in boys than in girls (*p < *0.01, cluster‐size corrected, all clusters > 324 voxels). Coordinates are Talairach coordinates of the peak voxel

Lobe	Region	BA	x	y	z	No. of voxels^a^	Cluster *t*
Occipital	Left lingual gyrus extending to middle occipital gyrus	17, 18	−22	−86	3	394	4.57
Parietal	Right inferior parietal lobule	40	41	−47	51	354	5.47
Parietal	Left precuneus extending to superior parietal lobule	7	−19	−71	45	1,092	4.74
Frontal	Right middle frontal gyrus extending to subgyral frontal lobe	6	32	−5	48	502	4.23
Occipital	Left middle occipital gyrus extending to middle temporal gyrus	18, 19, 39	−25	−89	18	1,194	4.96

Note

BA: Brodmann area; first region mentioned = region at the peak voxel; other regions arranged in order of decreasing size.

a Voxel size refers to the 1 × 1 × 1 mm^3^ resolution of the iso‐voxeled structural images.

**Figure 3:**
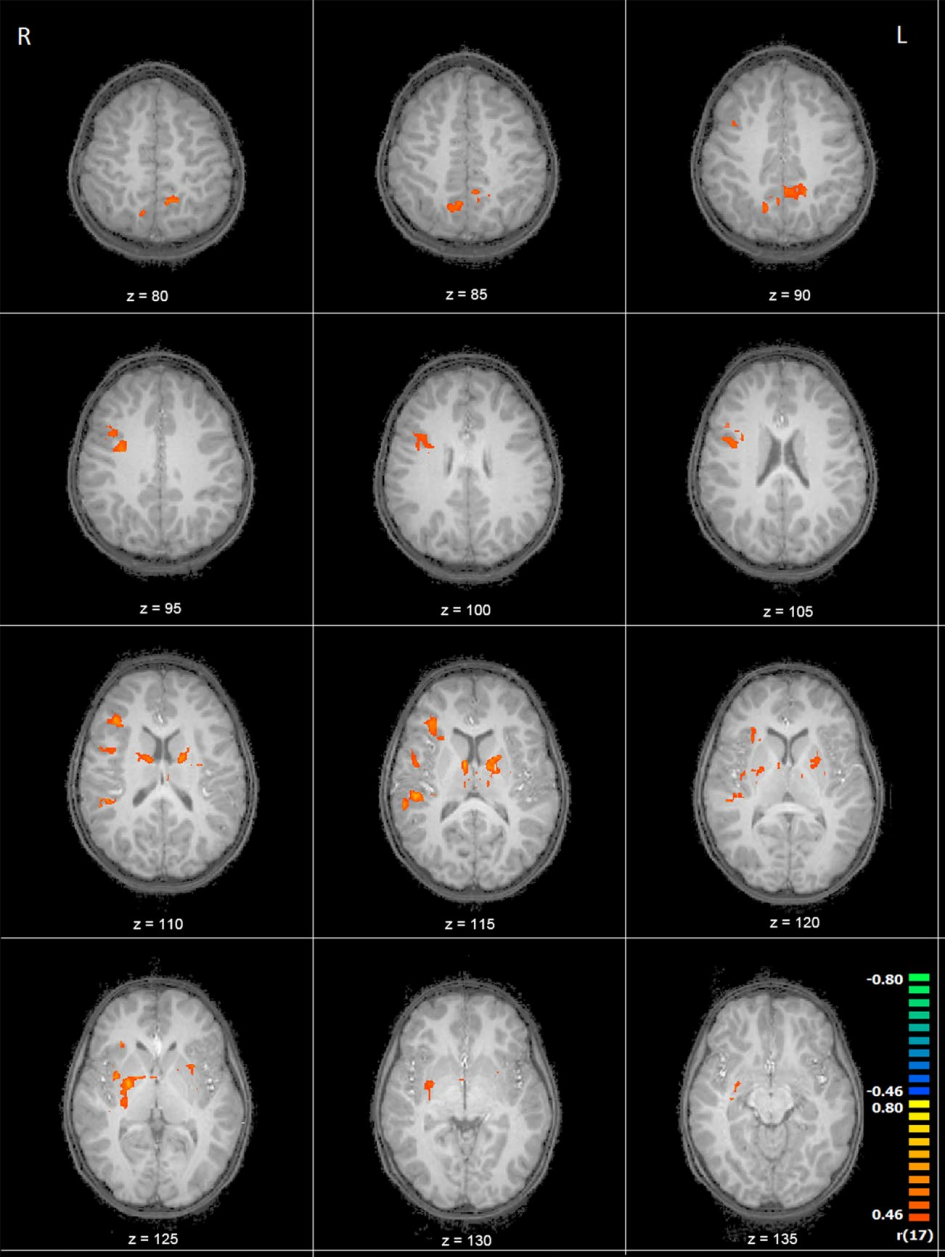
Transverse slices showing regions where activation increases during the invisible condition compared to the visible condition are negatively related to absolute alcohol per day in boys only (*z* = Talairach coordinate)

**Figure 4:**
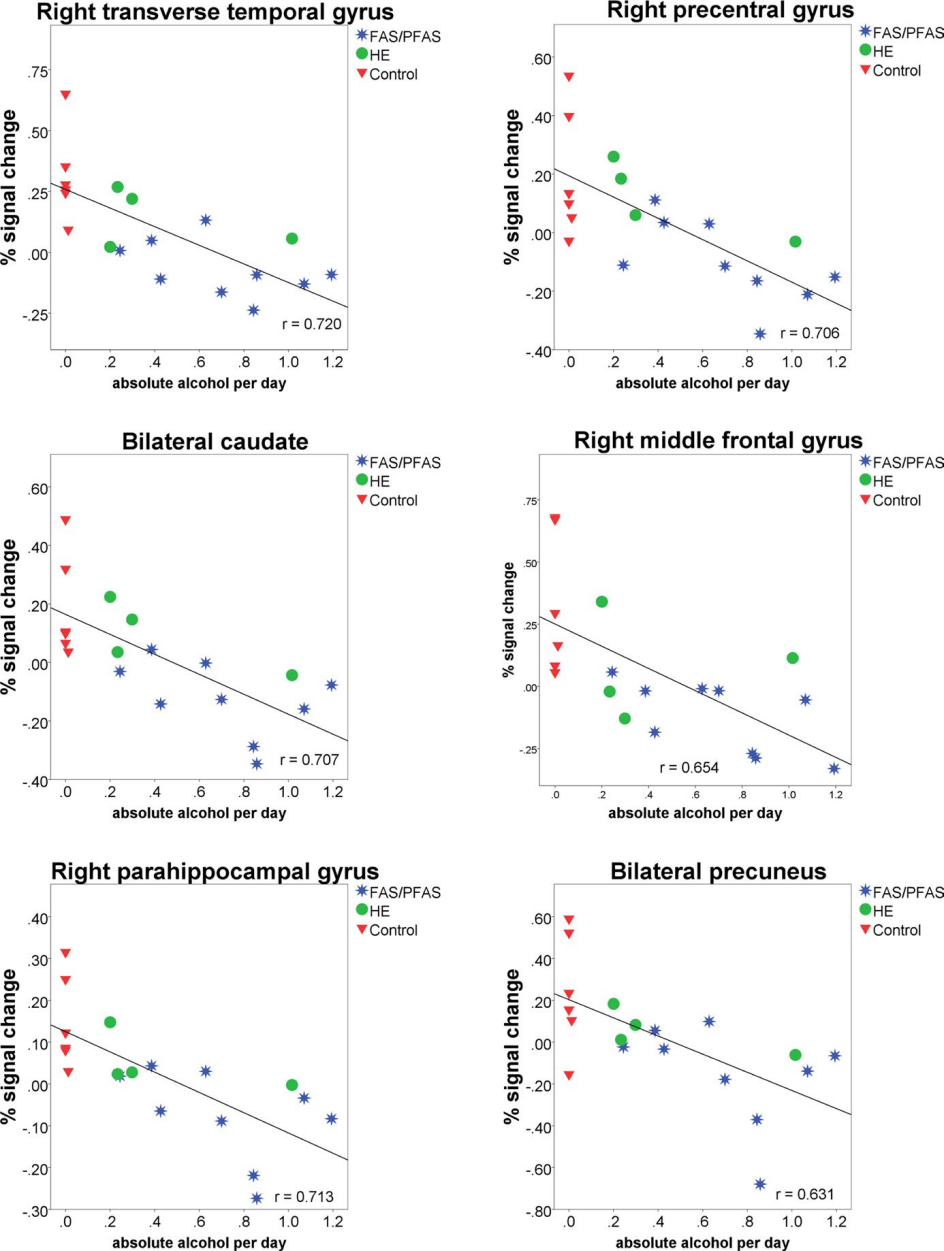
Relation of absolute alcohol per day to regional percentage signal change in boys

Because we observed differences in brain activation in boys and girls and because several of our previous imaging studies have shown that continuous alcohol measures are often more sensitive than diagnostic categorical measures (du Plessis, et al., 2014; Meintjes, et al., 2014; Woods, et al., 2015), we examined whether there were regions where differences in activation between the invisible‐ and visible‐target conditions were associated with levels of PAE within each sex separately. In girls, there were no regions where differences in activity were related to degree of alcohol exposure. In contrast, as shown in Table 6, in boys, increasing alcohol exposure was strongly associated with smaller activation increases between the invisible‐target and visible‐target conditions in the right parahippocampal gyrus, superior temporal gyrus, transverse temporal gyrus, precentral gyrus, inferior frontal gyrus, middle frontal gyrus, insula and claustrum, bilateral precuneus, caudate, thalamus and lentiform nucleus, and the left posterior cingulate (Figures 3 and 4). Additional analyses showed that the findings persisted after removing the controls (all *r*s < −0.60, all *p*s < 0.01).

**Table 6:** Regions where increasing alcohol exposure is associated with smaller activation increases *in boys* during the invisible condition compared to the visible condition (*p < *0.05, cluster‐size corrected, all clusters > 1,026 voxels). Coordinates are Talairach coordinates of the peak voxel

Lobe	Region	BA	x	y	z	No. of voxels^a^	*r* or ß^b^ (*p*)
Temporal	Right transverse temporal gyrus extending to superior temporal gyrus and insula	13, 41, 42	47	−26	12	1,041	−0.72 (0.001)
Frontal	Right precentral gyrus extending to inferior frontal gyrus, insula, and middle frontal gyrus	6, 9, 13, 44	32	−5	29	2,182	−0.71 (0.001)
Basal ganglia	Bilateral caudate extending to thalamus and lentiform nucleus	‐	−10	−2	15	2,247	−0.71 (0.001)
Frontal^c^	Right middle frontal gyrus extending to claustrum, insula, and inferior frontal gyrus	13, 46	38	28	18	1,082	−0.65 (0.002)
Limbic	Right parahippocampal gyrus extending to lentiform nucleus and claustrum	‐	23	−2	−9	1888	−0.71 (0.001)
Parietal	Bilateral precuneus extending to left posterior cingulate	7, 31	8	−53	45	2,100	−0.63 (0.004)

Note

AA: absolute alcohol; BA: Brodmann area; first region mentioned = region at the peak voxel; other regions arranged in order of decreasing size.

a Voxel size refers to the 1 × 1 × 1 mm^3^ resolution of the iso‐voxeled structural images.

b Pearson correlation or standardized regression coefficient (when adjusting for potential confounding) of mean % signal change in the cluster with AA/day.

c Adjusted for age at testing.

Analyses examining relations between control variables and differences in mean % signal change between invisible‐ and visible‐target conditions in each of the regions where associations of activation with level of alcohol exposure were found in boys, detected only one significant correlation: Child's age at scan was related to differences in activation in the cluster centered on the right middle frontal gyrus (*r* = 0.27, *p* = 0.08; for all other correlations, *p* > 0.20). After controlling for child's age in this region, the association with degree of alcohol exposure remained significant (Table 6).

Across FASD diagnostic groups, greater activation increases in boys in the bilateral precuneus and a large left frontal region during the invisible‐target condition compared to the visible‐target condition were associated with improved place learning as reflected both by shorter path lengths (Table 7a) and by a shorter latency (Table 7b) to the target. These correlations remained significant after controlling for alcohol exposure (all *p*s < 0.042). In girls, there were no regions where activation increases were associated with better performance.

**Table 7:** Regions where *in boys* smaller activation increases during the invisible condition compared to the visible condition were associated with (a) longer path lengths (*p* < 0.05, cluster‐size corrected, all clusters > 1,107 voxels) and (b) increasing latency (*p < *0.05, cluster‐size corrected, all clusters > 1,134). Coordinates are Talairach coordinates of the peak voxel

Lobe	Region	BA	*x*	*y*	*z*	No. of voxels^a^	Cluster correlation	Cluster effect size^b^
**(a) Regions associated with path length**								
Parietal	Bilateral precuneus extending to left cuneus	7, 31	−4	−65	39	1869	−0.61 (*p* = 0.005)	−0.48 (*p* = 0.035)
Frontal	Left superior frontal gyrus extending to medial frontal gyrus, middle frontal gyrus, and cingulate gyrus	6, 8, 9, 10, 32	−16	43	33	2,312	−0.59 (*p* = 0.008)	−0.45 (*p* = 0.042)
**(b) Regions associated with latency**								
Frontal	Left anterior cingulate extending to medial frontal gyrus, middle frontal gyrus, and superior frontal gyrus	6, 8, 9, 10, 24, 32	−4	19	24	3,370	−0.67 (*p* = 0.002)	−0.64 (*p* = 0.006)
Parietal	Bilateral precuneus extending to cuneus	7	8	−56	51	1520	−0.59 (*p* = 0.008)	−0.55 (*p* = 0.022)

Note

BA: Brodmann area; first region mentioned = region at peak voxel; other regions arranged in order of decreasing size.

a Voxel size refers to the 1 × 1 × 1 mm^3^ resolution of the iso‐voxeled structural images.

b Adjusted for absolute alcohol per day across pregnancy.

All findings were essentially unchanged when the analyses were rerun omitting the four children whose mothers used illicit drugs during pregnancy.

## 4 DISCUSSION

In this study, we detected no sex differences in behavioral performance. However, activation increases during the invisible‐target condition compared to the visible‐target condition were greater in boys than in girls in parietal regions, including the precuneus and the superior and inferior parietal lobules, as well as in frontal and occipital regions. In contrast, no regions showed greater activation increases during the invisible‐target condition in girls. In boys, PAE was associated with poorer place learning and reduced activation increases during the invisible‐target condition in the parahippocampal gyrus, precuneus, posterior cingulate, frontal and temporal lobes, caudate, insula, claustrum, lentiform nucleus, and thalamus, with most of these being right lateralized. Notably, better place learning in boys was associated with greater activation increases during the invisible‐target condition in a precuneus region similar to that showing PAE effects, as well as a large left frontal region. In girls, PAE was not associated with performance or activation differences between invisible‐ and visible‐target conditions in any regions, nor was better performance related to activation differences between conditions in any regions.

Although most studies report better allocentric navigational performance in males, including prepubertal children (Newhouse, et al., 2007), some using computer‐simulated versions of the MWM (Astur, et al., 1998, 2004; Burkitt, et al., 2007; Daugherty, et al., 2014; Driscoll, et al., 2005; Mueller, et al., 2008; Nowak, et al., 2014; Sneider, et al., 2015; van Gerven, et al., 2012; Woolley, et al., 2010) have found no sex differences in performance (Jacobs, et al., 1997, 1998; Sneider, et al., 2011). It is possible that inclusion of a probe trial, where the platform is removed unbeknownst to the participants, may have increased sensitivity in detecting sex differences in these studies as a male advantage has been demonstrated previously in probe trials even in the absence of performance differences in invisible‐target trials (Nowak, et al., 2014; Sneider, et al., 2015).

Despite the absence of sex differences in performance, our finding that no regions showed greater activation increases during invisible‐target conditions in girls than in boys, compared to greater increases in activity during invisible conditions in parietal, frontal, and occipital regions in boys, provides further evidence that males and females use different navigational strategies (Liu, Levy, Barton, & Iaria, 2011; Rodgers, Sindone, & Moffat, 2012; Sandstrom, Kaufman, & Huettel, 1998) and activate different brain regions (Sneider, et al., 2011) during place learning. Females tend to navigate egocentrically, using mainly landmark‐based cues, whereas males tend to navigate allocentrically, using mainly Euclidean information, such as direction, distance, gradient (slope of the floor), and geometry (Andersen, Dahmani, Konishi, & Bohbot, 2012; Astur, et al., 1998; Coluccia & Louse, 2004; Grön, et al., 2000; Moffat, Hampson, & Hatzipantelis, 1998; Nowak, Murali, & Driscoll, 2015; Sandstrom, et al., 1998; Saucier, et al., 2002; Woolley, et al., 2010).

Although effects of PAE on place learning have been reported in studies involving both males and females (Mattson, et al., 2010), at least three other studies have reported effects in males only—one, an animal study, in which only male rats showed impaired working memory in a T‐maze (Zimmerberg, et al., 1991); another in which only male rats were examined and which showed poorer and perseverative performance on the MWM by those moderately exposed to alcohol (Hamilton, et al., 2014); and the third, a human study that also did not include girls in which boys with FAS took longer paths to find a hidden platform (Hamilton, et al., 2003).

PAE has been shown to have a greater impact on spatial location memory than on object memory. For example, Uecker and Nadal (1996; 1998) showed impairment in memory for object location (but not in the recall of the objects themselves) in children with FASD, a finding replicated by Kaemingk and Tanner Halverson (2000). Poorer performance has also been demonstrated on the visual learning task from the wide range assessment of memory and learning (Sheslow & Adams, 1990), in which subjects are asked to remember the location of designs (Kaemingk, et al., 2003; Mattson & Roebuck, 2002). The findings that PAE appears to affect location memory specifically and that, in general, males rely more on spatial strategies for navigation may explain why their performance is impaired by PAE, whereas performance of females, who tend to rely more on landmarks (object memory), appears largely unaffected.

Notably, the right parahippocampal gyrus, where PAE was related to reduced activation, has been shown to mediate allocentric navigational strategies (Burgess, et al., 2002; Janzen & Weststeijn, 2007; Jordan, Schadow, Wuestenberg, Heinze, & Jäncke, 2004; Maguire, et al., 1998, 1999; McNaughton, et al., 1991; Nowak, et al., 2015; Parslow, et al., 2004), which tend to be employed more by males than by females (Nowak, et al., 2015; Sandstrom, et al., 1998; Saucier, et al., 2002). A number of other regions showing PAE effects in boys, including the precuneus (Frings, et al., 2006), posterior cingulate (Aggleton, Vann, Oswald, & Good, 2000; McNaughton, et al., 1991), caudate (Moffat, Kennedy, Rodrigue, & Raz, 2007), precentral gyrus (Rodriguez, 2010), and thalamus (Jordan, et al., 2004), are also associated with allocentric spatial memory.

It is possible that place learning in boys is more affected by PAE because they rely to a greater extent on brain regions more vulnerable to alcohol exposure. Notably, two of the regions affected by PAE in boys, the left precuneus and the right middle frontal gyrus (see Table 6), are among the regions showing greater activation increases during the invisible‐target condition compared to the visible‐target condition in boys than in girls (Table 5). Increased activation during invisible conditions in a similar region in the precuneus was also associated with better place learning performance in boys only (Table 7), suggesting that in boys, the use of the precuneus is an efficient strategy, whereas it is not in girls. To explore this idea further, we compared brain activation in unexposed boys and girls in those regions showing alcohol‐related impairment in boys. All the regions showing alcohol‐related alterations in activation in boys were activated more by unexposed boys than by unexposed girls. The regions preferentially activated by control girls may be less affected by PAE.

A limitation of this study was the relatively small sample sizes, especially those of the HE and control groups. Sample sizes were smaller than planned because about 28% of the children (*n* = 16) were excluded from the functional analysis due to excessive motion. It is possible that a larger sample might have revealed a relation between PAE and activation in girls. Another limitation is that we scanned the children only while they were passively learning the virtual environment and not while actively navigating through it. We followed this procedure because it is difficult for children to navigate with a joystick and remain still in the scanner at the same time—even during the passive task we had problems with excessive motion. Furthermore, our post‐scan navigation task did not include a probe trial to test learning. Performance on that trial may have been more sensitive to sex differences in performance and could have been used to examine differences in navigational strategies.

Future studies could include an egocentric version of the arena, as well as the allocentric version used here. It would be interesting to see whether PAE affects the performance and neural correlates of boys and/or girls in the egocentric version.

## 5 CONCLUSIONS

Girls and boys are known to use different navigational strategies. Our findings suggest that the use of these different strategies requires the involvement of different brain regions and that whereas the regions used by boys are susceptible to PAE damage, those used by girls appear to be relatively spared.

## CONFLICT OF INTEREST

The authors declare no competing financial interests.
